# Predicting Compressive Strength of Foamed Concrete Based on Anisotropy of the Pore Structure with the Influence of Pore Size and Shape Using X‑ray Computed Tomography

**DOI:** 10.1021/acsomega.6c00793

**Published:** 2026-03-25

**Authors:** Yanru Chen, Changyi Tang, Mingkai Cui, Long Sun, Ming Liu, Guoxing Sun

**Affiliations:** † 624082 Institute of Applied Physics and Materials Engineering, University of Macau, Macau SAR 999078, P.R. China; ‡ Zhuhai UM Science & Technology Research Institute, Zhuhai, Guangdong 519000, P.R. China; § Zhuhai Institute of Urban Planning & Design, Zhuhai, Guangdong 519000, P.R. China; ∥ Guangdong Huazheng Construction Co., Ltd., Zhuhai, Guangdong 519000, P.R. China; ⊥ Zhuhai Transportation Holdings Group Co., Ltd., Zhuhai, Guangdong 519000, P.R. China; # CCCC Third Harbour Consultants Macau Co., Ltd., Macau SAR 999078, P.R. China; g Fujian Provincial Key Laboratory of Terahertz Functional Devices and Intelligent Sensing, School of Mechanical Engineering and Automation, 12423 Fuzhou University, Fuzhou, Fujian 350108, P.R. China

## Abstract

In order to reveal the anisotropy of the pore structure and predict the compressive strength of foamed concrete based on its pore characteristics, X-ray computed tomography was used to characterize the pore structure. Empirical models were modified to correlate pore structure with compressive strength considering the anisotropy of the pore structure, pore size, and shape, based on the pore structure in the compression direction rather than the overall pore structure. For the first time, the compressive strength of foamed concrete was related to cross-sectional porosity, average pore diameter, and circularity in the compression direction. The pore size and shape were considered in modified models through a newly proposed pore size factor and pore shape factor, respectively. The modified Balshin model was found to be the most suitable for predicting the compressive strength of foamed concrete based on the anisotropy of the pore structure, along with the influence of the pore size and shape.

## Graphical abstract

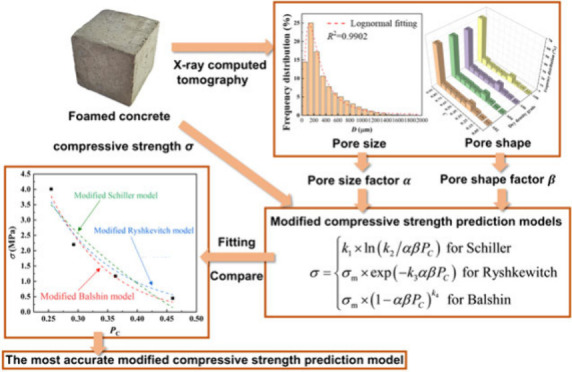

## 1 Introduction

Foamed concrete of a lightweight porous material created by mixing stable foam with slurry cementitious material has found extensive applications in modern engineering construction, due to its effective thermal insulation, favorable strength-to-weight ratio, excellent fluidity of fresh concrete, and reduced cement content.^1^ Foamed concrete can meet the requirements of different constructions by adjusting its density. Foamed concrete with a density lower than 600 kg/m^3^ is mostly used as the insulation layer for walls and floors, due to its high porosity that offers good insulation performance.^2,3^ Foamed concrete with a density ranging from 600 kg/m^3^ to 1200 kg/m^3^ can flow through narrow spacings and quickly fill into voids during long-distance transportation due to its high fluidity and low settlement, so it is often used for well backfilling and cavity filling.^4^ Foamed concrete with a density of 1200 kg/m^3^ is used for load-bearing components such as maintenance of road subbases and ground stabilization, due to its high strength and low cement usage.^5,6^ The pore structure, as the core element of the microscopic organization of materials, has a decisive influence on the macroscopic mechanical properties of various materials. This rule has been widely verified in cement-based materials, rocks, ceramics, and composite material systems.^7,8^ In rock mechanics, variations in pore connectivity, pore size distribution, and pore anisotropy have been shown to strongly govern strength degradation, crack initiation, and failure modes under compression, due to the stress concentration induced at pore boundaries.^9,10^ Similar effects have been observed in porous ceramics and metal–ceramic composites, where the evolution of pore morphology critically affects elastic modulus, fracture toughness, and long-term durability.^11,12^ Moreover, in various porous material systems, including ceramics and metallic biomaterials,^13,14^ density and porosity have been shown to strongly influence flexural strength, elastic modulus, and fracture toughness, as demonstrated in studies on porous ceramics and Ti6Al4V implants.^15,16^ These findings indicate that the density-dependent mechanical behavior observed in foamed concrete is consistent with the general mechanical response of porous solids. The mechanical properties of foamed concrete are significantly influenced by the pore structure,^17^ cementitious material, foaming agents, and their interactions.^18,19^ The pore structure is a critical determinant of the strength of foamed concrete,^20^ and it was reported that porosity had the most substantial adverse impact on compressive strength of foamed concrete.^21^ A more uniform distribution of more spherical pores of smaller sizes can lead to enhanced compressive strength^22–26^ by mitigating stress concentration.^27^ Among the various techniques used for assessing the structure of concrete, X-ray computed tomography (X-ray CT) has the advantage of nondestructively detecting internal closed pores in concrete.^28–32^ In contrast, mercury intrusion method (MIP),^33,34^ nitrogen physisorption test (NPT),^35^ nuclear magnetic resonance (NMR),^36–39^ and scanning electron microscope (SEM)^40^ are limited to open pores.^41^ X-ray CT enables the observation of larger samples compared to SEM^42^ and industrial camera photoanalysis,^43^ and can provide higher accuracy than acoustic wave detection.^44–47^ Tian et al.^48^ employed X-ray CT to monitor the corrosion and cracking in steel-reinforced concrete and obtained the spatial mapping of corrosive agents within the samples. Zhang and Zhu^49^ utilized X-ray CT to characterize the features and distribution of fibers and pores in cement-based materials.

The compressive strength of porous materials (e.g., porous sintered alumina and zirconia, gypsum pastes, and porous metal ceramic) can be predicted by the discrete element method, finite element method, and mathematical formulas.^50–52^ The parameter calibration of discrete element method relies on detailed microscopic structure characterization and has a high computational cost.^53^ The quality of mesh division in the finite element method has a significant impact on the calculation results, and the modeling workload is considerable when simulating complex pore structures.^54^ The mathematical formula prediction method is the fundamental approach for studying the strength of porous materials, and it is simple in form and has clear physical meaning, which can be extended and applied to different kinds of concrete (e.g., foamed concrete^55^). The strength of cellular concrete can be predicted by considering the water-cement ratio, density, and theoretical strength of matrix at zero porosity,^56^ effect of aging,^57^ and different additives^58^ based on the Balshin model.^52^ The Ryshkewitch model^51,59,60^ was found to be more applicable to geopolymer foamed concrete than the Balshin model^61^ and more suitable to predict the compressive strength of concrete of low porosity than the Schiller model^50^ and the Balshin model.^62^ The Schiller model and Ryshkevitch model were found to be almost identical to predict the strength of cement mortar, except for the material of extremely high or low porosity.^63^ Almost the same strength can be obtained by the Ryshkewitch model and Balshin model for foamed concrete of porosity in the range of 0.37–0.72.^64^ Both the structure and strength of concrete are anisotropic,^65,66^ particularly for foamed concrete of a porous material with uneven structure. The existing models are subject to significant prediction error since the proposed models were based on the porosity of the overall pore structure without considering the effects of pore size and shape on strength.

In the current study, X-ray CT was used to characterize the cross-section and 3D pore structure of foamed concrete, whose compressive strength was predicted by considering the pore structure in the compression direction rather than the overall pore structure. An empirical model was proposed for predicting the compressive strength of foamed concrete, considering the cross-sectional porosity, pore size, and shape (i.e., average pore diameter and circularity) in the compression direction.

## 2 Materials and Characterization

### 2.1 Preparation and Characterization of Foamed Concrete

Foamed concrete specimens of four different densities were designed, and the mixing proportions required for producing concrete of 1 m^3^ are listed in Table 1. According to the Chinese standard JGJ/T 341-2014, the formulations for different dry density grades were designed by adjusting the relative proportions of cement paste and preformed foam to achieve the target dry densities. In this study, the initial mixture proportions were based on the formulation reported in ref 67, and the final formulations listed in Table 1 were obtained through trial-mix optimization following an experience-based mixture-design approach. The physical foaming agent powders (Yifa Building Materials Technology Co., Ltd., Jurong, Jiangsu, China) stabilized by nanoparticles^67^ were dispersed and stirred in water at a ratio of 0.33 wt % until fully dissolved. The fresh foam generated by the foam generator had a density of approximately 50 kg/m^3^ (measured by collecting a known foam volume in a graduated cylinder and dividing the mass by the volume). Foaming agents used in foamed concrete generally fall into three categories: physical foaming agents, which generate foam through mechanical agitation; chemical foaming agents, which release gas through chemical reactions; and protein-based foaming agents, which produce highly stable foam from hydrolyzed proteins. The foaming agent adopted in this study belongs to the category of physical foaming agents and was selected due to its stability and good compatibility with cementitious materials, reported in ref 67. Chemical compositions of the ordinary Portland cement (Type I 42.5) used in this study are listed in Table 2. Cement paste prepared by mixing cement and water at a speed of 120 r/min for 2 min and the fresh foam generated by the foam generator were combined and stirred for 2 min and then were poured into cubic molds of 100^3^ mm^3^. The foamed concrete specimens were demolded after 48 h and cured under standard conditions (temperature of 20 ± 2 °C, relative humidity >90%) for different periods (i.e., 7 days, 14 days, and 28 days). After curing of 28 days, the specimens were dried at 60 °C for 72 h until unvarying weight. It should be noted that the foamed concrete consists only of water, Portland cement, and preformed foam, and all mixtures share the same cement type, water–cement ratio, curing conditions, and preparation procedure. Since the foam does not participate in hydration, the hydration reactions are governed by the cement and water, and thus the types of hydration products formed, including low-density silicate hydrate (HD C–S–H), high-density silicate hydrate (HD C–S–H), calcium hydroxide (CH), and unhydrated cement (UC), are expected to be identical across different dry density grades. The primary distinction among mixtures lies in the volume fraction of pores introduced by the foam, while the phase assemblage and the volume fractions of hydration products remain essentially unchanged. If XRD or thermogravimetric analysis were performed, all mixtures would exhibit the same characteristic phase signatures with only slight variations in peak intensity or mass-loss magnitude due to the dilution effect associated with increased air content, rather than any fundamental differences in hydration chemistry. Therefore, this study focuses on the pore structure, while hydration phase composition is considered consistent across all density grades. The dry density of concrete can be calculated by the unvarying weight divided by the volume of the concrete and is listed in Table 1. In this study, the dry density refers to the envelope density, calculated from the mass after drying to constant weight and the geometric volume of the 100 mm cubic specimens, following Chinese standard JGJ/T 341-2014. For clarity, the envelope density differs from the true density and skeletal density of the material: the true density represents the density of the solid matrix excluding all pores, while the skeletal density excludes only open pores but includes closed pores. Since all mixtures use the same cementitious matrix and only vary in th efoam content, the true density and skeletal density remain essentially constant among different density grades. In contrast, the envelope density varies significantly with foam dosage and therefore serves as the relevant density index for characterizing foamed concrete. The formulations listed in Table 1 are reported according to this envelope density definition. The compressive strength of foamed concrete was measured by the compressive test at 1 kN/s on an MTS (Jinan Time Shijin Testing Machine Co., Ltd., Jinan, Shandong, China). The fluidity of fresh foamed concrete and the water absorption were measured in accordance with the Chinese standard JGJ/T 341-2014. The hydration rates of the foamed concrete of different dry density grades were monitored using TAM air isothermal calorimetry (TA Instruments), in which the heat-release rate of each mixture was continuously recorded for 72 h to capture the evolution of the main hydration peak under standardized conditions.

**Table 1:** Formulations of Four Different Samples^a^

dry density grade	cement (kg)	water (kg)	foam (kg)	dry density (kg/m^3^)
A02	167	94	28.2	233.9 ± 6
A04	333	186	23.4	429.5 ± 5
A06	500	280	18.3	584.3 ± 6
A08	667	374	13.5	785.8 ± 8

a Note: the grades of dry density are according to Chinese standard JGJ/T 341-2014.

**Table 2:** Compositions (wt %) of Cement

CaO	SiO_2_	Al_2_O_3_	Fe_2_O_3_	MgO	SO_3_	K_2_O	L.O.I.
55.95	19.89	8.17	4.97	1.69	3.25	0.64	3.69

In accordance with previous studies on foamed concrete, the wet density of the fresh mixture was used to calculate the foam porosity *P*_f_. As reported in refs 68 and 69 , the pore system formed in the fresh state governs the subsequent development of pore morphology in hardened foamed concrete, making the use of wet density consistent with the physical definition of initial foam porosity. In contrast, the dry density is strongly affected by water evaporation, hydration reactions, and drying-induced deformation during curing and thus does not represent the pore volume originally introduced by the foam. The foam porosity *P*_f_ of foamed concrete, which can be regarded to be the total porosity in foamed concrete, can be calculated as

1 $$P_{f}=1-\rho /\rho_{0}$$

where ρ and ρ_0_ are wet densities of fresh foamed concrete and base mixture measured before curing.

Figure 1 shows the influence of the dry density on the fluidity and water absorption of foamed concrete. Quadratic dependence of fluidity and water absorption on dry density can be seen, and the fluidity is positively related to dry density, while water adsorption is negatively related to the dry density, consistent with refs 70 and 71 , Fluidity is related to the uniformity of the fresh mixture, which is negatively affected by the foam content. Because foam, as a component with a larger size relative to cement particles and lower fluidity than the cement paste, will isolate the cement paste and reduce the continuity of the cement paste as a liquid phase. The fluidity of foamed concrete is mainly influenced by its density, while the water-cement ratio, admixtures, and additives also have an impact. In practical engineering, if the fluidity does not meet the requirements and the density cannot be changed, it is possible to improve the fluidity by increasing the water-cement ratio and adding admixtures and additives. Adding an appropriate amount of fly ash and mineral powder can increase the fluidity while reducing costs. The water reducers can enhance fluidity, while the accelerators can reduce fluidity. The foamed cement of a lower density has more space (i.e., open and interconnected pores) for water storage due to its higher porosity, larger pore sizes, and more microcracks, resulting in higher water absorption. Moreover, the foamed cement of a lower density has more capillary pores,^72^ further enhancing its water absorption. The water absorption is an intuitive indicator for measuring the resistance of foamed concrete to the erosion of harmful substances, such as water and salt, from the outside. Foamed concrete with low water absorption has more closed and independent pore structures, which can effectively block the transmission paths of water and harmful substances, thereby ensuring long-term stability in harsh environments such as dampness, freeze–thaw, and dry–wet cycles. Foamed concrete with high water absorption has a large number of open pores or interconnected pores, which may be destroyed after experiencing freeze–thaw cycles or dry–wet cycles, resulting in the decline in the strength of foamed concrete.

**Figure 1:**
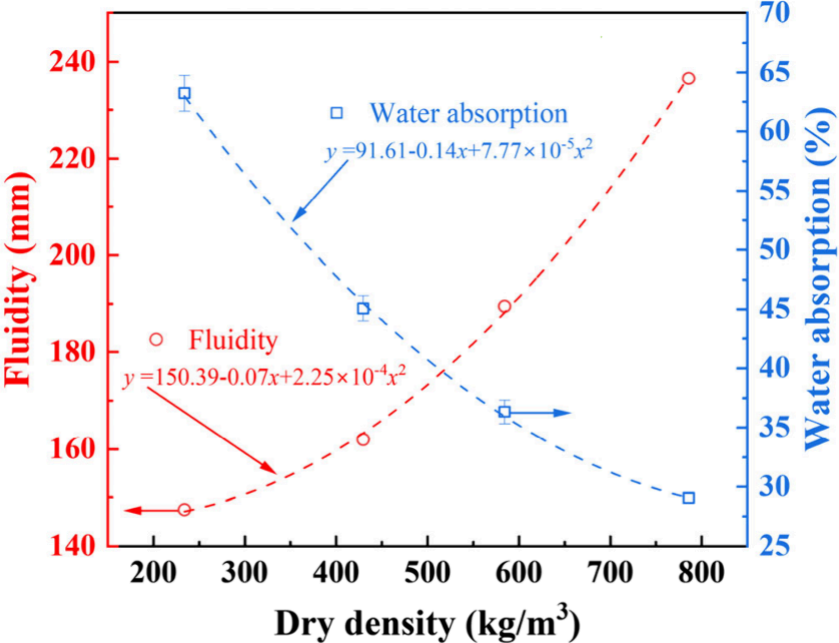
Influence of dry density on fluidity and water absorption of foamed concrete.

### 2.2 Characterization of Pore Structure of Foamed Concrete by CT

#### 2.2.1 CT Image Processing Method

The structure of foamed concrete was characterized by X-ray CT scanning (Sanying Precision Instruments Co., Ltd., Tianjin, China) at a voltage of 200 kV and a maximum power of 320 W on WorX microfocus X-ray and industrial CT system. The spatial resolution of the scanned image is 60.15 μm/voxel, and the number of pixels is 1900 × 1900. The raw images from X-ray CT scanning were processed by Avizo software for image analysis and 3D reconstruction to assess the pore structures of specimens,^41^ as shown in Figure 2. The raw grayscale images were initially denoised through the Gaussian filtering algorithm. The regions with higher and lower gray values represent greater (or brighter) and lower (or darker) densities (or areas), respectively. The bright regions indicate the matrix, and the dark regions denote the pores. The pore structure was determined by a binary processing method (i.e., threshold segmentation) assigning each pixel value as either 0 (pore) or 1 (solid), and the separation operation is carried out to reduce the error. The threshold used for binarizing the CT images was selected by visually identifying the grayscale inflection between the pore phase and the cement matrix, which is a commonly adopted practice in pore-structure characterization of foamed concrete due to the clear contrast between air voids and solid regions.^73^ To verify the adequacy of the selected threshold, the pore boundaries and pore diameters obtained from CT segmentation were directly compared with those measured from the corresponding optical microscopy images of the same specimen region (see Figure 2 for the same pore characterized by CT and optical microscopy), and the two sets of measurements showed good agreement. This cross-validation indicates that the chosen threshold reliably captures the actual pore geometry. Ultimately, the 2D images of varying depths were stacked to reconstruct the three-dimensional porous structure of 1000 cross sections.

**Figure 2:**
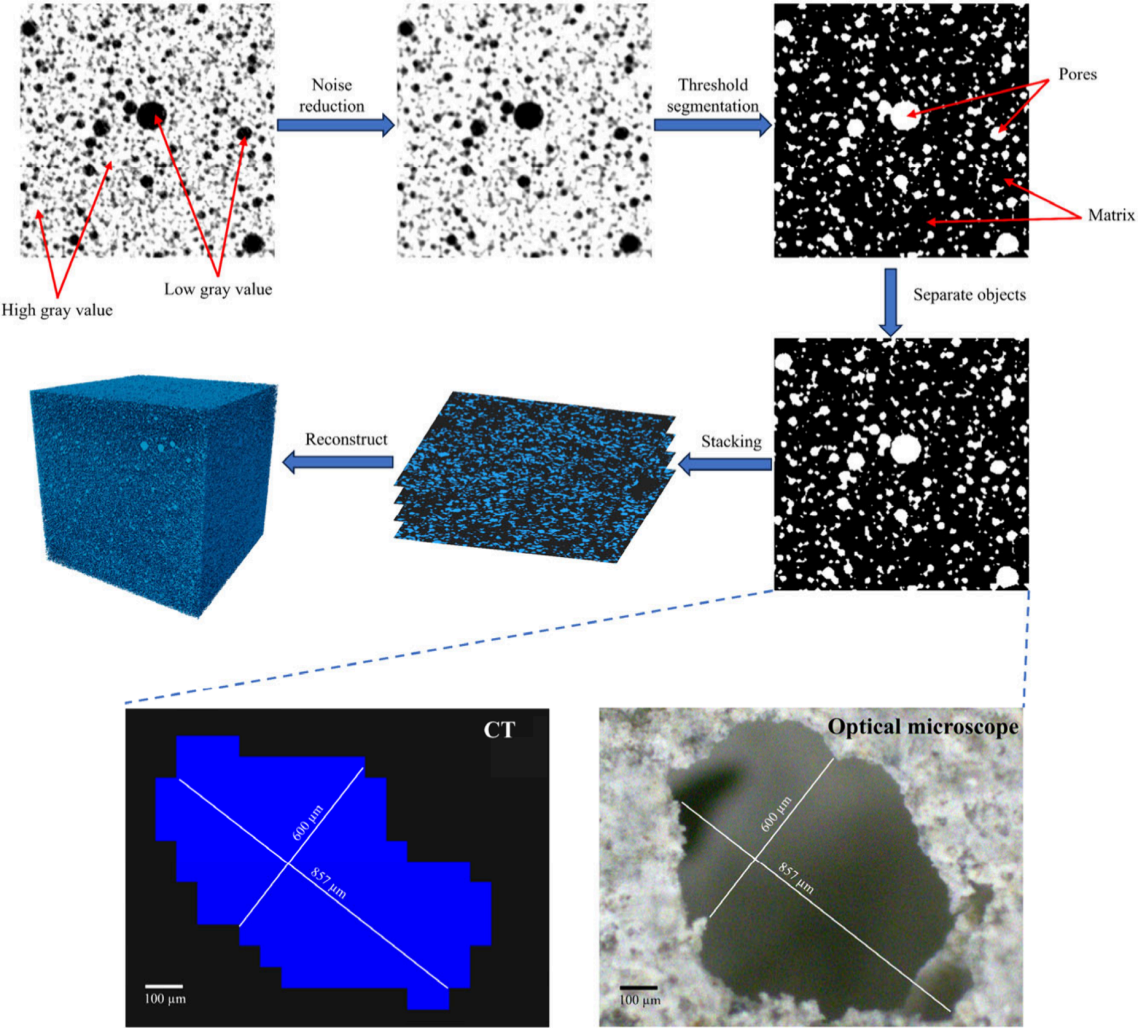
Image processing procedure of the raw images by Avizo software with comparison of the same pore determined by CT and an optical microscope.

#### 2.2.2 Characterization of Pore Size and Shape

The cross-sectional porosity *P*_s_ can be calculated by the area of pores divided by the cross-sectional area of foamed concrete on the processed image of a certain depth. The cross-sectional porosities at different depths can be obtained, resulting in the average value *P*_0_ based on all cross-sectional images. In order to investigate the anisotropy of foamed concrete, values of *P*_0_ obtained along three orthogonal axial directions (i.e., *x*, *y*, and *z* directions with *y* and *z* directions being parallel to compression and pouring directions, respectively; see Figure 3) were compared.

**Figure 3:**
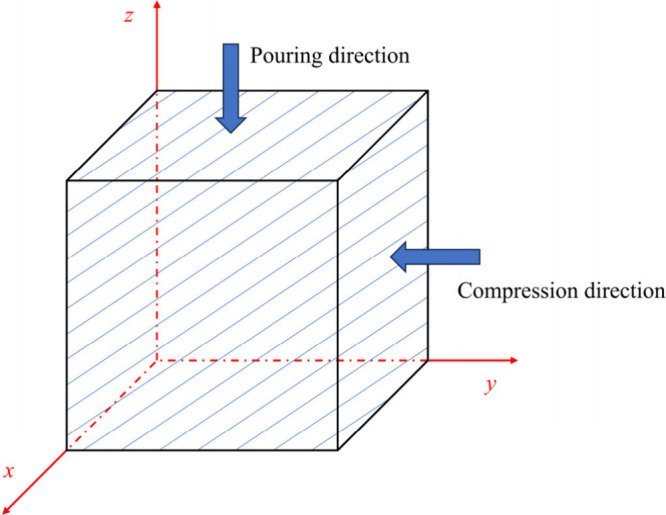
Three-dimensional Cartesian coordinates of cubic foamed concrete with *y* and *z* axis parallel to compression and pouring directions, respectively.

The pore size and shape of a pore can be characterized by the pore diameter *D* and circularity factor *C*, respectively, on the cross section^74^

2 $$D_{i}=\sqrt{4A_{i}/\pi },C_{i}=4\pi A_{i}/L_{i}^{2}$$

where the subscript *i* denotes an individual pore; *A* and *L* are the area and circumference of the pore, respectively. Based on geometric principle, 0 < *C* ≤ 1, and a larger *C* indicates a more circular pore with *C* = 1 representing a perfectly circular pore. In order to investigate the anisotropy of foamed concrete, *D*_s_ (i.e., the average values of *D*_*i*_) and *C*_s_ (i.e., the average values of *C*_*i*_) on the cross section were obtained and compared along three orthogonal axial directions (i.e., *x*, *y* and *z* directions). *D*_0_ and *C*_0_ are the average values of *D*_s_ and *C*_s_, respectively, based on all of the cross-sectional values at different depths along the compression direction (i.e., *y* direction).

## 3 Results and Discussion

### 3.1 Pore Structure of Foamed Concrete

#### 3.1.1 Pore Diameter Distribution Analysis

Figure 4 shows the distribution of the pore diameter for foamed concrete of different dry density grades based on 10 cross-sectional images extracted from each of the three samples at equal intervals in the compression direction, with curve fitting by the probability density function of logarithmic normal distribution:

3 $$f=y_{0}+A/\left(\sqrt{2\pi }\sigma x\right)\;\exp\left[-{\left(\ln\;x-\mu \right)}^{2}/2\sigma^{2}\right]$$

where *y*_0_ and *A* are the fitting parameters; μ and σ are the mean and standard deviation of the logarithm of the pore diameters, and the mathematical expectation *E* and variance *S* can be obtained by

4 $$E=\exp\left(\mu +\sigma^{2}/2\right),S=\left[\exp\left(\sigma^{2}\right)-1\right]\;\exp\left(2\mu +\sigma^{2}\right)$$

**Figure 4:**
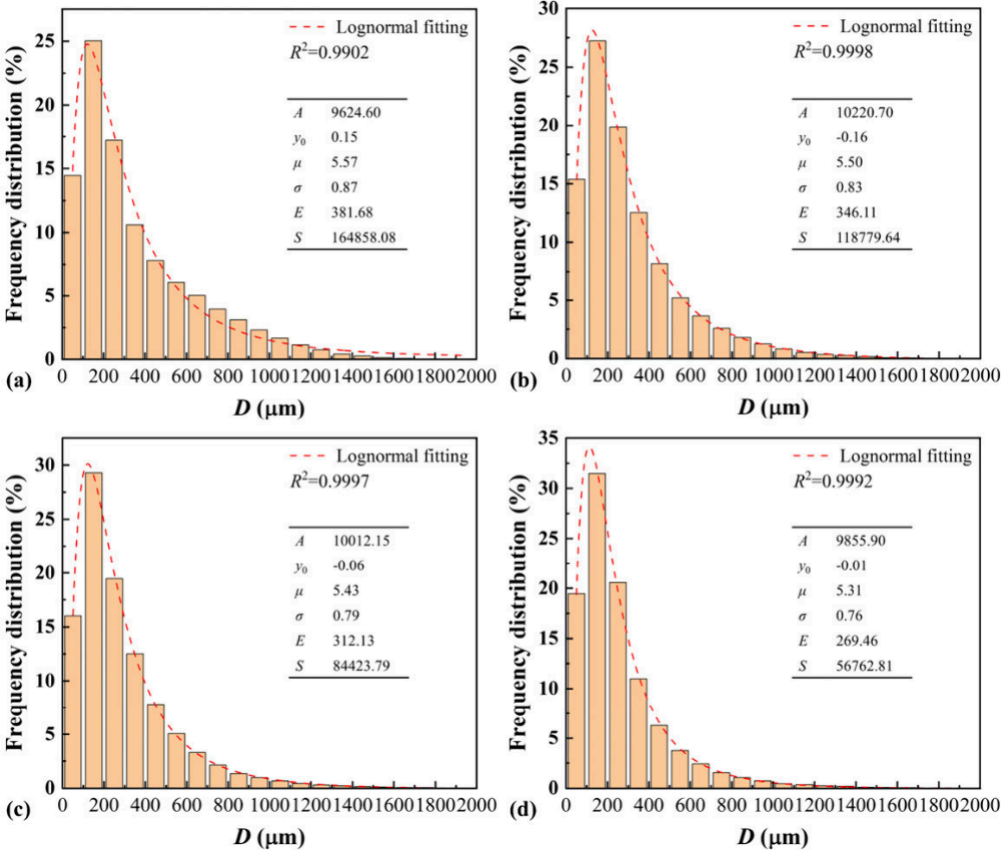
Pore diameter distributions of foamed concrete of different dry densities (the insets show the fitting parameters): (a) A02; (b) A04; (c) A06; and (d) A08.

For A02, A04, A06, and A08, the pore diameters are concentrated in the range of 100–200 μm, accounting for 25.03%, 27.24%, 29.29%, and 31.47%, respectively. This indicates that the distribution of pore diameters becomes more uniform as dry density increases due to the lower proportion of cement in foamed concrete of a lower density, which can result in a slower hardening of cement, and thus the bubbles cannot be adequately fixed and tend to gather and merge into larger ones more frequently, resulting in larger pores of uneven distribution. *R* ^2^ > 0.99 signifies that the pore diameter of foamed concrete follows a nonstandard logarithmic normal distribution, consistent with the previous studies.^75,76^ As the dry density increases, the axis of symmetry of the distribution shifts to the left, which can be seen by the decrease in μ; see the insets of Figure 4. The distribution width becomes narrower, which is indicated by the decrease in σ, and mathematical expectation *E* and variance *S* of the pore diameters become smaller.

Figure 5 presents the statistical parameters of pore diameter *D* of foamed concrete for different dry densities based on 10 cross-sectional images at equal intervals in the compression direction. The influence of dry density on statistical parameters of pore diameter of foamed concrete is quantitatively revealed for the first time: the average *D*_a_, median *D*_n_, and mode *D*_m_ of pore diameter *D* all decrease in an almost linear way with the increase in dry density, which is reasonable by noting that a larger dry density is associated with a smaller overall pore size. *D*_a_ > *D*_n_ > *D*_m_ for each specimen conforms to the right skewed distribution, which belongs to the logarithmic normal distribution, corroborating the applicability of eq 3 for curve fitting the distribution of pore diameter of foamed concrete.

**Figure 5:**
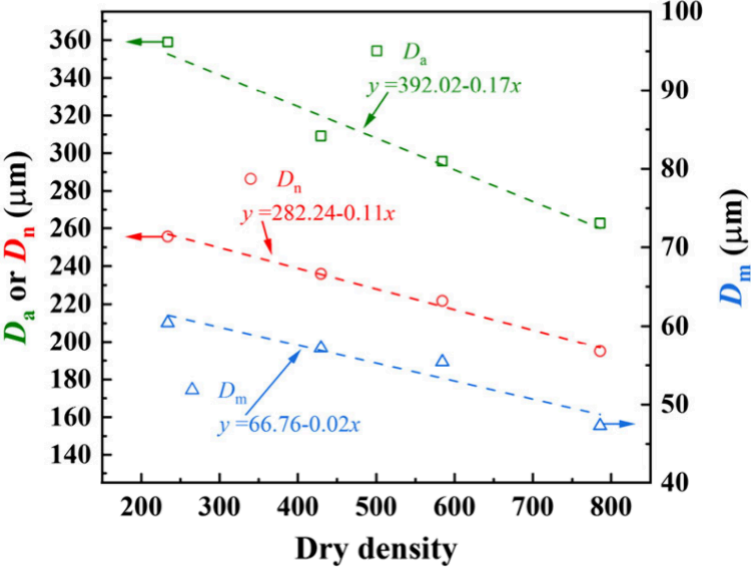
Influence of dry density on statistical parameters (i.e., the average *D*_a_, median *D*_n_, and mode *D*_m_) of the pore diameter *D* of foamed concrete.

#### 3.1.2 Pore Circularity Distribution Analysis

Figure 6(a) shows the distribution of pore circularity *C* for foamed concrete of different dry density grades obtained from 10 CT slices extracted from each of the three samples. *C* shows a bimodal distribution rather that the unimodal distribution reported in the previous study.^23^ The bimodal distribution of pore circularity can be attributed to the distinct mechanisms governing bubble evolution during the preparation of foamed concrete. Highly spherical pores (i.e., high *C*) primarily originate from stable, well-formed foam bubbles generated during the prefoaming process, which retain their shape when uniformly dispersed in the cement paste. In contrast, irregularly shaped pores (i.e., low *C*) arise from partial coalescence, deformation, and occasional bursting of bubbles during mixing, especially in regions with high local shear stresses. The primary (>56%) and secondary (∼10%) concentrations lie within the ranges of 0.9–1.0, and 0.3–0.4, respectively, indicating that more than half of the pores are nearly circular. Figure 6(b) presents the statistical parameters of pore circularity *C* of foamed concrete for different dry densities based on 10 cross-sectional images extracted from each of the three samples at equal intervals in the compression direction. A significant increase in *C*_a_ and *C*_n_ can be seen as the density increases from 584.3 ± 6 kg/m^3^ to 785.8 ± 8 kg/m^3^, while *C*_m_ increases as the density increases under small densities and deceases under large densities. *C*_m_ and *C*_n_ are similar and larger than 0.9, consistent with the primary concentration being larger than 50%. Values of *C*_a_ are smaller than those of *C*_m_ and *C*_n_, and are larger than 0.75, indicating that the pores of very irregular shapes occupy a small portion, and most pores have almost circular shapes.

**Figure 6:**
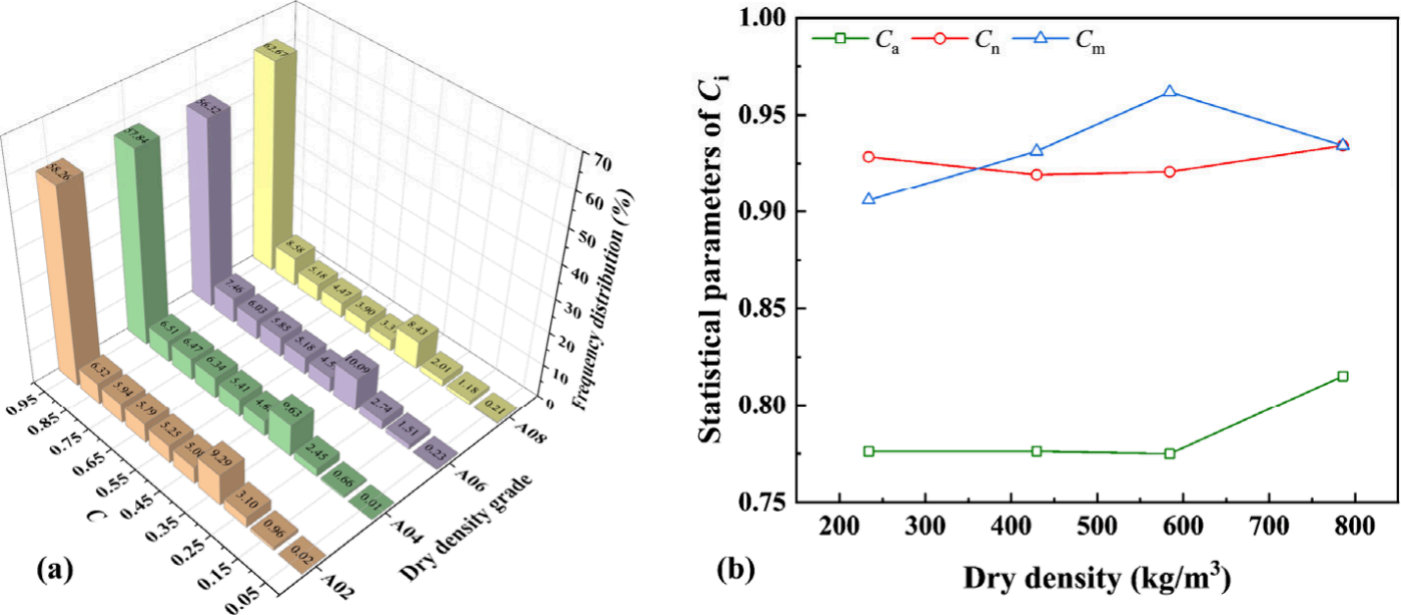
Statistical analysis of pore circularity *C* for foamed concrete of different dry density grades (A02, A04, A06, and A08): (a) distribution of *C* and (b) influence of dry density on statistical parameters (i.e., the average *C*_a_, median *C*_n_, and mode *C*_m_) of pore circularity *C* of foamed concrete.

Figure 7 shows the correlation between *C* and *D* for A08 (see SI.1 in Supporting Information for publication for those of A02, A04, A06). Figure 7(a) shows two distinguished regions on *C* vs *D* map: for small *D* (i.e., the left region of the red dashed line), as *D* increases, a larger *D* is associated with a greater variety of *C* and a higher probability of lower *C*; for large *D* (i.e., the right region of the red dashed line), as *D* increases, a large *D* is associated with a fewer variety of *C* and a higher probability of lower *C*. Therefore, a smaller pore is more likely to have a more spherical shape. Although the fresh foam before mixing with the cement paste is nearly spherical due to low surface tension,^23^ pores of various shapes (e.g., interconnecting pores appearing as long wedges, ovals, and gourds) with low circularity *C* occur for pores of a large pore diameter *D* due to foam aggregation, bursting, and fusion during mixing. The nearly negative correlation between *C* and *D* in foamed concrete, especially for pores of large *D*, is consistent with the previous study.^74^ Figure 7(b) presents the percentage of pores of *C* > 0.9 within different ranges of pore diameter *D*. Pores of *C* > 0.9 can only occur for *D* < 1200 μm. Pores of *D* in the range of 100–200 μm have the highest proportion of pores of *C* > 0.9. The percentage of pores of *C* > 0.9 for *D* of 0–100 μm is smaller than that for *D* of 100–200 μm. The proportion of high *C* (i.e., *C* > 0.9) decreases as *D* increases for *D* > 200 μm.

**Figure 7:**
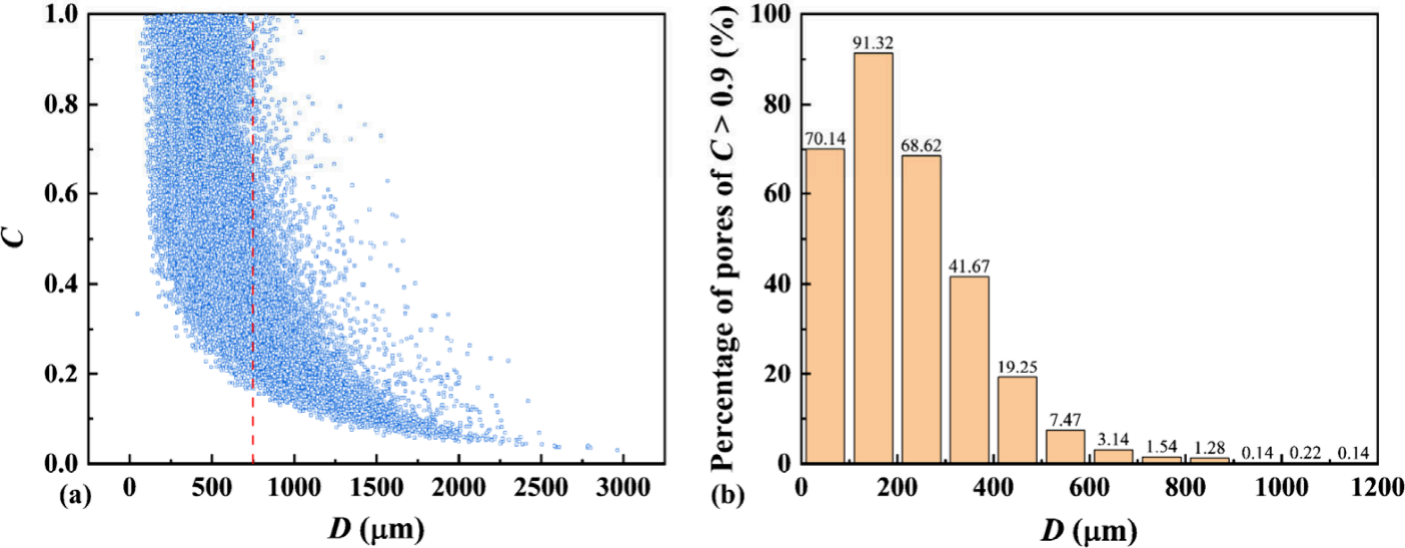
Correlation between pore diameter *D* and circularity *C* for A08: (a) *C* vs *D* map (the red dash line divides the pores into two parts); and (b) the percentage of pores of *C* > 0.9 within different ranges of pore diameter *D*.

#### 3.1.3 Anisotropic Structure of Foamed Concrete

Figure 8 and Figure 9 respectively show the cross-sectional images of different dry densities of foamed concrete obtained from X-ray CT, as well as three-dimensional (3D) pore structures, which are taken from one of the three parallel samples for illustration. The black regions (i.e., pores) in Figure 8 become less as the density increases, demonstrating that the porosity increases with density. In Figure 8(a), the uneven, slightly dented edges of A02, the foamed concrete with lowest density, can be attributed to its reduced early age strength and slower setting due to low cement content, which makes the mixture more sensitive to deformation under surface tension and bubble coalescence during the fresh state. Similar observations have been reported in previous studies,^77^ where early age shrinkage and delayed matrix formation caused local deformation of low-density foamed concretes. The pore structure of the foamed concrete can be assessed by pores of different sizes, which can be extracted and reconstructed by Avizo software. The pores of different sizes are not uniformly distributed within the foamed concrete, which can be explained by noting the difficulty in complete mixing of the cement paste with the foam due to the difference in density. The superlarge pores (>10 mm^3^), which are represented by yellow regions and predominantly appear on the specimen surfaces, can be clearly seen for the foamed concrete (i.e., A02) of the smallest density of 233.9 ± 6 kg/m^3^; become scarce for the foamed concrete (i.e., A04) of the second smallest density of 429.5 ± 5 kg/m^3^; and can be hardly seen for the foamed concrete (i.e., A06 of 584.3 ± 6 kg/m^3^ and A08 of 785.8 ± 8 kg/m^3^) of the two largest densities. The air is prone to be trapped at the edges of the cubic mold during pouring, resulting in significantly large voids and the formation of superlarge pores mainly at the edges of the cubic specimen after hardening. As the density increases, more purple regions, which represent small pores (<0.01 mm^3^), can be seen, and the green regions, which represent relatively large pores (i.e, 0.01–10 mm^3^) become fewer. Therefore, the increase in the density of foamed concrete is associated with a reduced proportion of large pores and an enlarged proportion of small pores.

**Figure 8:**
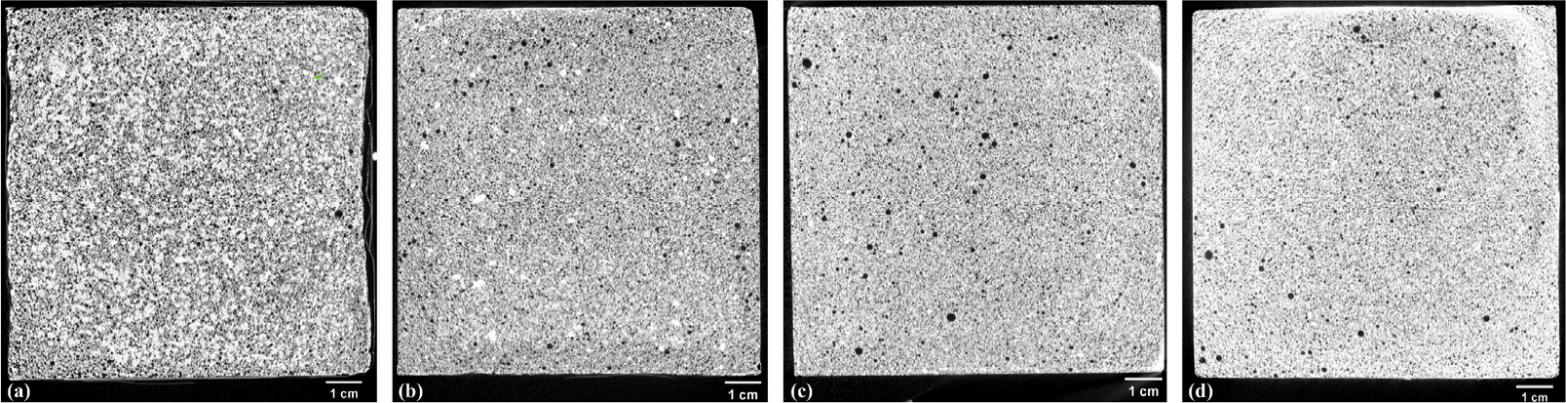
Cross-sectional images of foamed concrete of different dry densities obtained from X-ray CT: (a) A02; (b) A04; (c) A06; (d) A08. The black regions represent pores.

**Figure 9:**
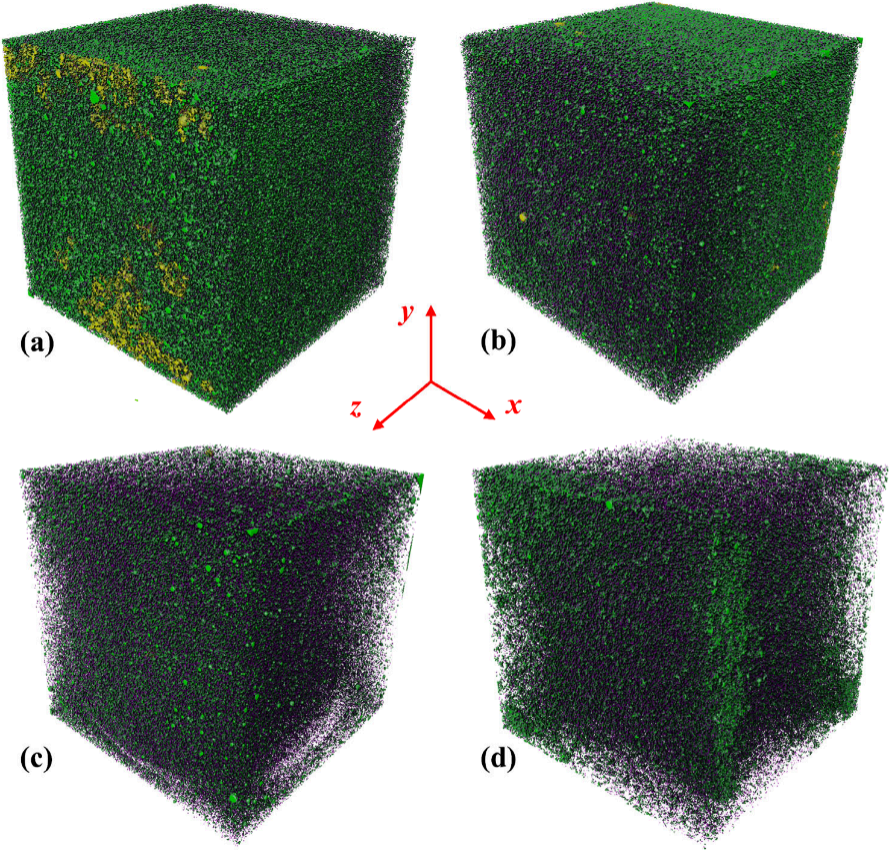
3D pore structures of foamed concrete (cubic shape of 100^3^ mm^3^) of different dry densities: (a) A02; (b) A04; (c) A06; and (d) A08. Purple regions represent pores smaller than 0.01 mm^3^, green regions represent relatively large pores from 0.01 mm^3^ to 10 mm^3^, and yellow regions represent superlarge pores larger than 10 mm^3^. Pouring direction is along the *z*-axis.

Figure 10 compares the variations of *P*_s_, *D*_s_ and *C*_s_ on the cross section at different depths in the *x*, *y*, and *z* directions of A08, which are averaged from three parallel samples at the same depth (see SI.2 in Supporting Information for publication for those of A02, A04, A06). The anisotropy of the pore structure of foamed concrete is evidenced by the different variations and average values of *P*_s_, *D*_s_, and *C*_s_ in the *x*, *y*, and *z* directions. This is due to the nonuniform distribution of foam in the pouring direction, resulting in fluctuations in porosity, pore diameter, and circularity, as foam floats up under the influence of gravity. The foam porosity *P*_f_ of the foamed concrete calculated by eq 1 is larger than the average values of *P*_s_ in the *x*, *y*, and *z* directions. Since the compressive strength of foamed concrete is mainly related to the structure, it is essential to connect the compressive strength with the pore structure in the compression direction rather than the overall pore structure (i.e., *P*_f_ that is used to predict compressive strength of foamed concrete) of foamed concrete.

**Figure 10:**
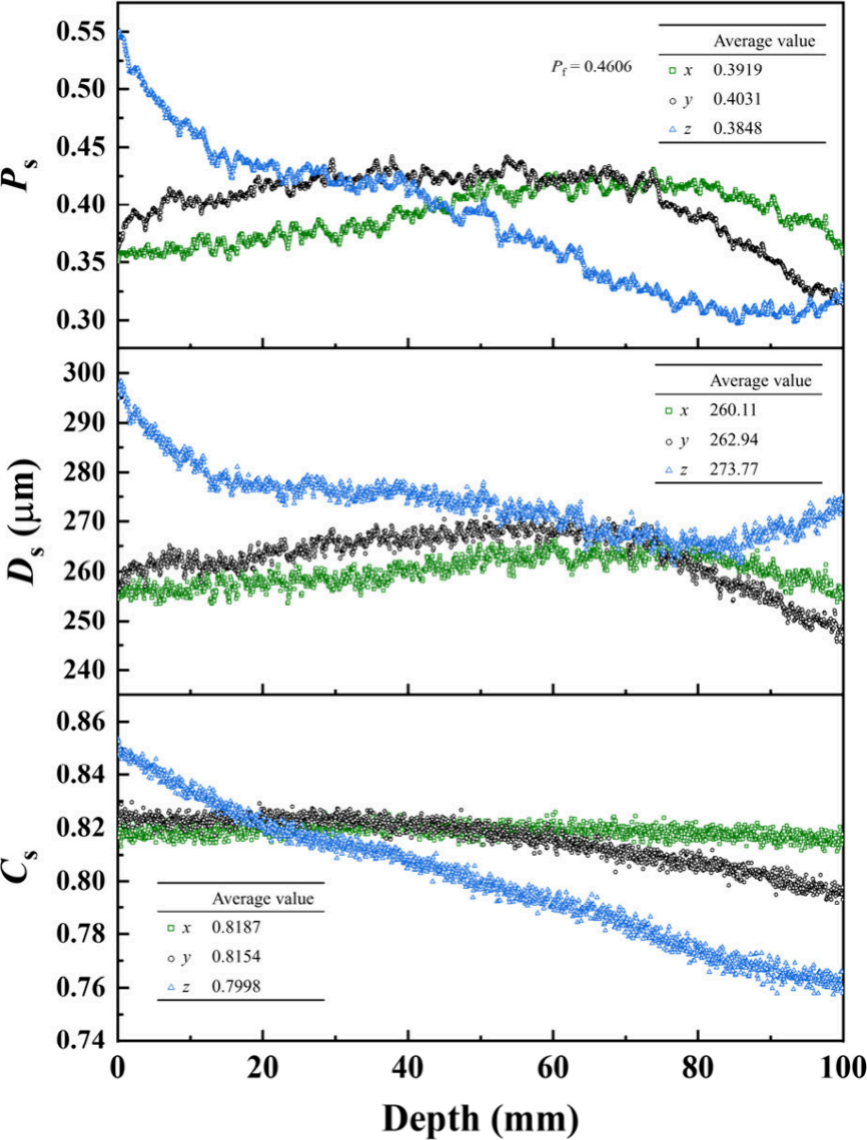
Variations of cross-sectional porosity *P*_s_, average pore diameter *D*_s_, and average circularity *C*_s_ in the *x*, *y*, and *z* directions of foamed concrete of A08. The average values of *P*_s_, *D*_s_ and *C*_s_ in per directions and the foam porosity *P*_f_ calculated by eq 1 are listed in the insets.

Figure 11 exhibits the average values (i.e., *P*_0_, *D*_0_, and *C*_0_) of *P*_s_, *D*_s_ and *C*_s_ in the compression direction (i.e., *y* direction) of foamed concrete of different dry densities. Both *P*_0_ and *D*_0_ show a negative linear relationship with dry density, and *C*_0_ has a positive linear correlation with dry density, since the pores of foamed concrete of higher density tend to have smaller values of porosity and diameter, and the shape of pores becomes closer to circular. Smaller pores are more beneficial for resisting compression than large pores, since the number of pores increases with a more uniform distribution and more circular shapes (i.e., a larger *C*) as the pore diameter becomes smaller, resulting in a more uniform distribution of stress around the pores with less stress concentration.^27^

**Figure 11:**
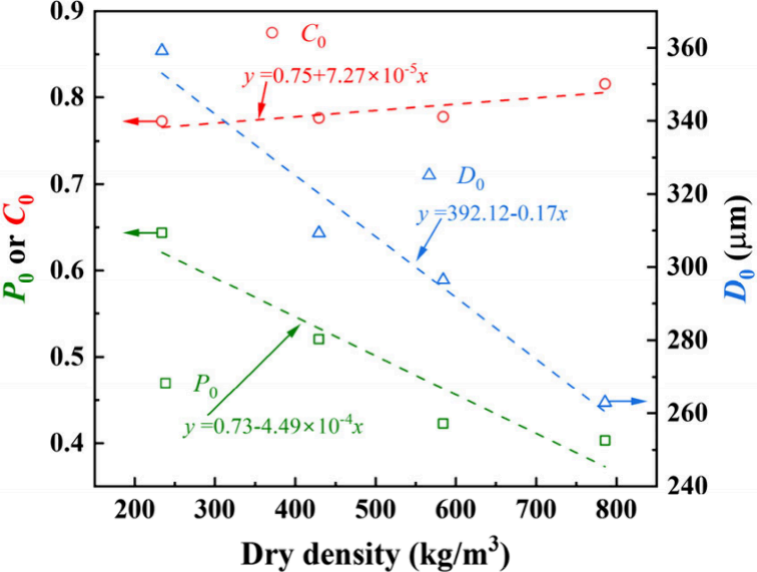
Average values (i.e., *P*_0_, *D*_0_, and *C*_0_) of *P*_s_, *D*_s_, and *C*_s_ in the compression direction (i.e., *y* direction) of foamed concrete for different dry densities.

### 3.2 Compressive Strength and Stress–Strain Curve

Figure 12 shows the results of the compression test of foamed concrete of different densities. Figure 12(a) displays the compressive stress–strain curve of foamed concrete of different densities at 28 days, which can be divided into three stages, described as follows:

**Figure 12:**
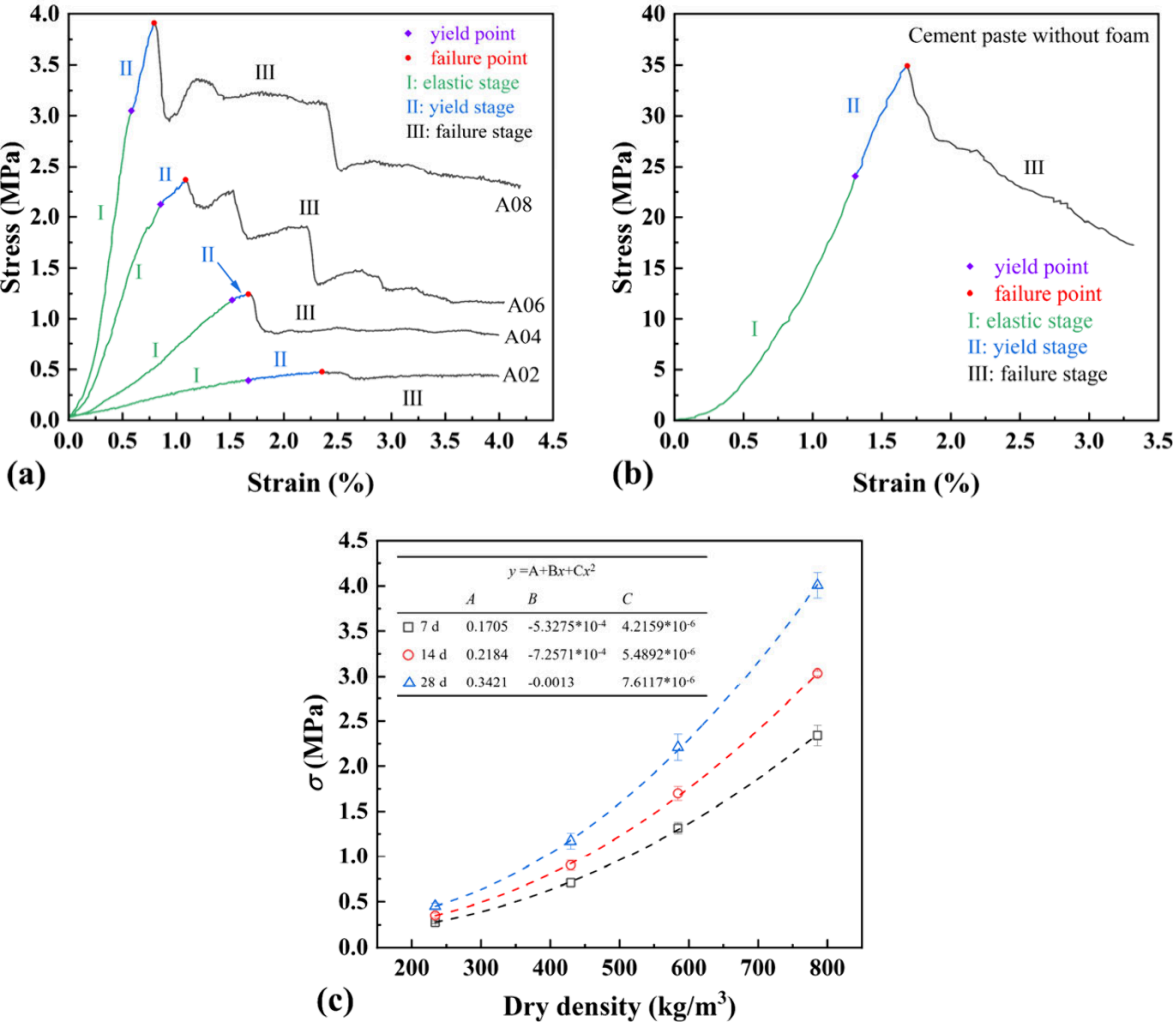
Compressive stress–strain curves of (a) foamed concrete of A02, A04, A06, and A08 at 28 days, (b) cement paste without foam at 28 days, and (c) influence of dry density on the compressive strength of foamed concrete at different ages (the curve fitting parameters are listed in the inset).

Stage I is the elastic stage. The stress increases linearly with strain with the slope representing the effective elastic modulus *E*_eff_. The specimen bears the load without cracking and stores elastic energy stress via the elastic deformation of the matrix with pore walls. Stage II is the yield stage. The onset of yield stage (i.e., yield point), where the stress is defined as effective yield strength σ_s_,^78^ is indicated by the decrease in the slope of stress vs strain, and slight fluctuation of stress caused by microcracks, which contributes to the permanent plastic deformation. Stage III is the failure stage. As stress gradually rises to the peak (i.e., failure point), where the stress is compressive strength σ, the specimen enters the failure stage, and stress tends to decline as strain increases. As the load increases, some pores can be destroyed due to stress concentration at weak points within the pore walls, subsequently leading to formation of microcracks, which can propagate and diminish the bearing capacity of the material. It can be seen that *E*_eff_, σ_s_, and σ all increase with dry density of foamed concrete, consistent with the results in previous studies.^79^

Figure 12(b) shows the compressive stress–strain curve of the cement paste without foam at 28 days, and three stages (i.e., elastic, yield, and failure stages) like those of foamed concrete can also be determined. The peak stress of the cement paste without foam is much larger than those of foamed concrete. The curve of the cement paste without foam is smoother with fewer fluctuations due to its uniform structure, resulting in less local failure. The failure stage (i.e., Stage III) shows typical brittle failure, characterized by a rapid decrease in stress after the peak, consistent with the result in the previous study.^80^ Comparison of compressive stress–strain curves between the cement paste without foam and foamed concrete reveal that the failure mode of foamed concrete under compression is ductile with postpeak stress decreasing in steps and maintaining a high level as the strain increases. The postpeak stress–strain curves demonstrate that foamed concrete possesses good ductility and the potential to absorb impact energy, endowing foamed concrete with extension of contact time, reduction in impact force upon collision, high impact resistance, and suitability for serving as a buffer material in applications such as the protection of a bridge pier against collision and engineered material arresting system (EMAS).^81^ The energy absorption of foamed concrete mainly relies on the large number of pores within it. When the impact energy exceeds the elastic limit of foamed concrete, the material dissipates energy by generating cracks and compressive deformation. During the process of the pore walls being damaged and compressed, impact energy is consumed. Compared with cement paste without foam that cannot undergo significant deformation, foamed concrete can be compressed and deformed due to the porous structure, which can prolong the contact time of the impact force and realize the energy absorption.

Figure 12(c) shows the influence of the dry density on the compressive strength σ of foamed concrete at different ages with the curve fitting by quadratic functions. The compressive strength of foamed concrete at different ages is consistent with the results in the previous study,^67^ and increases with dry density due to the reduction in porosity of cement-based material, and a quadratic relationship is found, for the first time, to be applicable to express the effect of the dry density on the compressive strength at different ages. A steeper curve for more ages implies that the effect of density on compressive strength becomes more significant over time. σ for a larger dry density increases by a larger extent with the increase in age, which can be explained by noting that the incorporation of foam can decrease dry density and delay the hydration process of the cement.^82^ The reduction in dry density results from the substantial increase in the entrained air volume, which decreases the amount of solid cementitious matrix per unit volume, as also described in ref 67. Previous studies have shown that the surfactants and stabilizers contained in foaming agents can adsorb onto the surfaces of cement grains, inhibit particle–particle contact, and impede ion exchange in the pore solution, thereby slowing down the hydration reactions.^82,83^ Moreover, the increased porosity introduced by the foam reduces the connectivity of the aqueous phase surrounding the cement particles, further delaying hydration kinetics. As the dry density of foamed concrete increases, the strength development can be accelerated by a higher cement content; see Table 1 for the cement content, and more hydration products. Both the compressive strength and the percentage increase over age increase with the dry density of foamed concrete. To investigate the hydration behavior of foamed concrete with different densities, TA Instruments was used to measure the heat flow of fresh foam concrete with different dry densities, as shown in Figure 13. Mixtures with a lower foam content (e.g., A08 and A06) exhibited an earlier and higher main hydration peak, indicating a faster hydration rate and greater cumulative heat release. In contrast, the main peak of the mixture with a higher foam content, such as A02, shifts significantly to the right, and the peak intensity decreases, suggesting that the addition of foam slows down the hydration process due to the increased separation between cement particles and the dilution of active phases.

**Figure 13:**
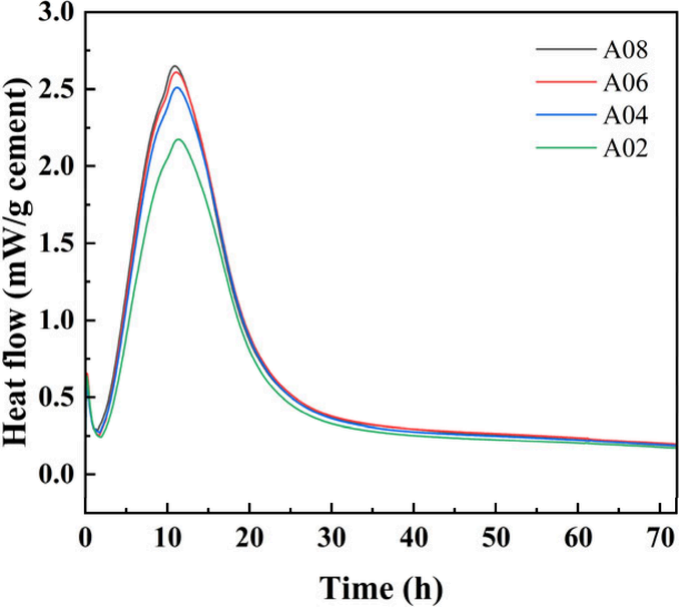
Heat flow of fresh foam concrete with different dry densities.

## 4 Models Predicting Compressive Strength Based on Pore Structure

### 4.1 Influence Factors of Pore Size and Shape

Based on the models proposed by Ryshkewitch,^51^ Schiller,^50^ and Balshin,^52^ the compressive strength σ of foamed concrete can be correlated with the overall pore structure (i.e., the foam porosity *P*_f_) of the foamed concrete:^68,69,84^

5 $$\sigma =\{\begin{array}{l} k_{1}\times \ln\left(k_{2}/\chi \right)\;\mathrm{for Schiller}\\ \sigma_{m}\times \exp\left(-k_{3}\chi \right)\;\mathrm{for Ryshkewitch}\\ \sigma_{m}\times {\left(1-\chi \right)}^{k_{4}}\;\mathrm{for Balshin} \end{array}$$

where χ is *P*_f_ and can be modified by considering effects of anisotropy, pore size and shape; σ_m_ is the compressive strength of the matrix of the foamed concrete (i.e., the cement paste without foam prepared under the same water to cement ratio of 0.56) and is a constant (i.e., σ_m_ = 35.61 MPa in the current work); *k*_1_, *k*_2_, *k*_3_, and *k*_4_ are empirical constants obtained from regression analysis of experimental data; *k*_2_ has the same unit as σ, while *k*_2_, *k*_3_, and *k*_4_ are dimensionless.

Since pores in foamed concrete are not evenly distributed in different directions, as observed in Figure 9 and Figure 10, and the compressive strength of foamed concrete is more related to the pore structure in the compression direction than the overall pore structure,^66^ χ should be replaced by *P*_0_, which is the average value of *P*_s_ in the compression direction (i.e., *y* direction). Effects of pore size and shape on the compressive strength of foamed concrete, which are neglected in eq 5, can also be considered by simple modification of *P*_0_ by the pore size factor α and pore shape factor β in the compression direction:

6 $$P_{c}=\alpha \beta P_{0},\alpha =D_{0}/D_{e},\beta =1/C_{0},D_{e}=\sqrt{4AP_{0}/n\pi }$$

where *P*_c_ is the modified *P*_0_ accounting for both the effects of pore size and shape, and χ in eq 5 should be replaced by *P*_c_ when the influence of both pore size and shape are taken into consideration; *D*_0_ and *C*_0_ are the average values of *D*_s_ and *C*_s_ in the compression direction (i.e., *y* direction in the current study) of foamed concrete; *D*_e_ is the effective pore diameter; *n* is the average value of the number of pores on the cross sections in the compression direction; *A* (= 10^4^ mm^2^ in the current study) is the cross-sectional area of the specimen; and AP_0_ represents the average value of cross-sectional area of pores. As for the hypothetical specimen with the same *P*_0_ as the actual specimen but a uniform distribution of pores of the same size and perfect circularity, *D*_0_ = *D*_e_, and *C*_0_ = 1, resulting in α = β = 1. Both α and β are dimensionless and serve as relative comparison regarding pore size and shape, respectively, with the hypothetical specimen as a baseline. The introduction of α and β is based on well-established micromechanical observations regarding foamed concrete. Previous experimental and numerical studies have demonstrated that the increase in pore diameter leads to higher stress concentration around the mid-diameter region of the voids, causing earlier crack initiation and a reduction in compressive strength.^85^ Similarly, irregular or nonspherical pore shapes have been shown to intensify stress concentration and further weaken the load-bearing capacity of foamed concrete, with mixtures containing less spherical pores exhibiting noticeably lower relative strength. These findings indicate that pore size and pore shape play both direct and measurable roles in the mechanical behavior of foamed concrete. Accordingly, α and β were formulated as dimensionless parameters that quantify the deviation of actual pore geometry from an idealized state with uniform pore size and perfectly circular pores. Incorporating these two factors into the strength-prediction model therefore provides a physically meaningful way to account for the effects of pore size and shape that are not explicitly included in the original models in eq 5. Values of α and β of foamed concrete of different dry density grades are listed in Table 3. Both α and β decrease with the increase in the dry density, suggesting that the pores tend to become smaller and more circular as the density increases.

**Table 3:** Pore Size Factor *α* and Shape Factor *β* of Foamed Concrete of Different Dry Density Grades

dry density grade (dry density)	α	β
A02 (233.9 ± 6 kg/m^3^)	0.5520	1.2940
A04 (429.5 ± 5 kg/m^3^)	0.5418	1.2882
A06 (584.3 ± 6 kg/m^3^)	0.5383	1.2858
A08 (785.8 ± 8 kg/m^3^)	0.5150	1.2263

### 4.2 Comparison of Different Models with Different Porosities

Figure 14(a) and (b) shows the influence of different porosities, i.e., *P*_f_ and *P*_c_, on the compressive strength of foamed concrete, respectively, with curve fitting by eq 5. Although compressive strength of foamed concrete can be predicted well by *P*_f_ with different models, the best fitting occurs for the Balshin model based on *P*_c_, demonstrating the suitability of the Balshin model to predict the compressive strength of foamed concrete when more factors such as the anisotropy of the pore structure, pore size, and shape are considered. To quantitatively evaluate the predictive performance of the different modified empirical models, the root-mean-square error (RMSE) and mean absolute error (MAE) were calculated based on the measured and predicted compressive strengths, listed in Table 4. For the modified Balshin model, the RMSE and MAE were 0.2142 and 0.1812 MPa, respectively, indicating excellent agreement with the experimental results. The error values of the other models were higher, confirming that the modified Balshin model provides the most accurate representation of the relationship between the pore-structure parameters and compressive strength. These quantitative results further support the conclusion that the modified Balshin model is the optimal predictive model among those examined. The Schiller model requires two fitting parameters of unclear physical significance, while only one fitting parameter is required for the Ryshkewitch or Balshin model. Moreover, both Ryshkewitch and Balshin models require the same target property of a reference material (i.e., compressive strength of the cement paste without foam that is the matrix of the foamed concrete in the current work), endowing the Ryshkewitch and Balshin models with more physical background than the Schiller model. The influence of the pore structure on compressive strength of foamed concrete follows a power law in a better way than an exponential trend, and thus the Balshin model is preferable to the Ryshkewitch model. Therefore, a modified Balshin model based on the pore structure in the compression direction, influence of pore size, and shape is proposed to predict the compressive strength of foamed concrete as

7 $$\sigma =\sigma_{m}{\left(1-\alpha \beta P_{0}\right)}^{k}$$

**Figure 14:**
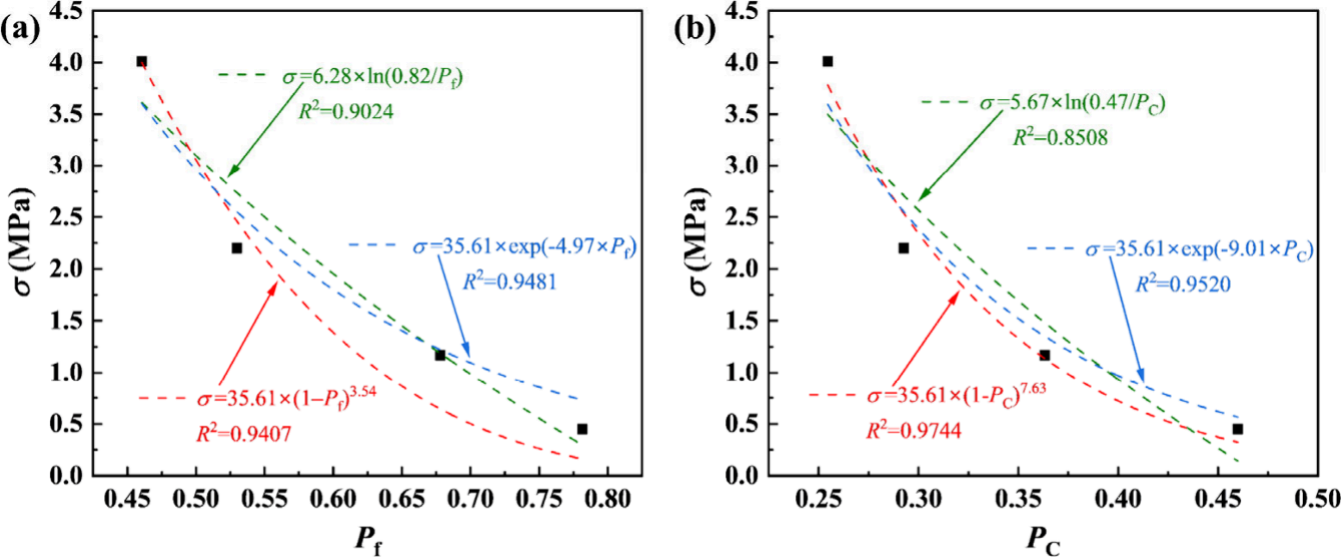
Influence of the pore structure on the compressive strength of foamed concrete with curve fitting by eq 5, in which χ is represented by (a) *P*_f_, and (b) *P*_c_.

**Table 4:** RMSE and MAE Based on the Measured and Predicted Compressive Strengths from Different Modified Empirical Models

modified empirical models	RMSE	MAE
σ = 5.67 × ln(0.47/*P*_C_) modified Schiller model	0.4225	0.4096
σ = 35.61 × exp(−9.01 × *P*_C_) modified Ryshkewitch model	0.2933	0.2662
σ = 35.61 × (1 – *P*_C_)^7.63^ modified Balshin model	0.2142	0.1812

It should be noted that the compressive-strength prediction model developed in this study is established under standard curing conditions, which represent the conventional basis for determining the characteristic strength of foamed concrete in engineering practice.^65,66,85^ Environmental influences such as freeze–thaw cycles, temperature fluctuations, and moisture variations typically involve additional deterioration mechanisms that belong to the domain of durability studies and are therefore beyond the intended scope of the present work. The proposed model is thus applicable to strength prediction under standard curing, while its extension to nonstandard environmental conditions may be addressed in future research focusing on long-term durability performance.

## 5 Conclusions

The pore structure of foamed concrete of different dry densities was characterized by X-ray CT, revealing the anisotropy of the pore structure by the variations of the average values of porosity, pore diameter, and circularity on the cross section in different directions. A smaller dry density of foamed concrete was associated with larger pores, a larger porosity, a wider range of pore size distributions, and a decrease in pore circularity. Compressive tests showed that foamed concrete possessed greater ductility and a higher potential to absorb impact energy compared to the cement paste without foam, and the compressive strength at different ages declined as the dry density decreased. Ryshkewitch, Schiller, and Balshin models were modified by considering the effects of anisotropy of the pore structure with the pore size and shape on the compressive strength of foamed concrete. The anisotropy of the pore structure was considered by the average of the cross-sectional porosity in the compression direction; pore size and shape were considered by newly proposed pore size factor and pore shape factor, respectively. The modified Balshin model was found to be most suitable to predict compressive strength of foamed concrete based on the anisotropy of the pore structure with the influence of pore size and shape.

## Supplementary Material



## Notes

Pore diameter *D* and circularity *C* of foamed concrete of A02, A04, and A06 (Figure S1); cross-sectional porosity *P*_s_, average pore diameter *D*_s_, and average circularity *C*_s_ in the *x*, *y* and *z* directions of foamed concrete of A02, A04, and A06 (Figure S2) (PDF)

## Notes

N23 University of Macau, Avenida da Universidade, Taipa, Macau, China
