# Supplementary material for: Predicting Compressive Strength of Foamed Concrete Based on Anisotropy of the Pore Structure with the Influence of Pore Size and Shape Using X‑ray Computed Tomography
Source: ACS Omega. 2026 Mar 25;11(13):21232–45. doi: 10.1021/acsomega.6c00793 (PMC13063038; doi:10.1021/acsomega.6c00793)
Supplement: Supplementary file 1 [file ao6c00793_si_001.pdf]

# Supporting Information for Publication

## Predicting compressive strength of foamed concrete based on anisotropy of pore structure with influence of pore size and shape using X-ray computed tomography

Yanru Chen [a], Changyi Tang [c], Mingkai Cui [d, e], Long Sun [f], Ming Liu [g, *], Guoxing Sun [a, b, **]

[a] *Institute of Applied Physics and Materials Engineering, University of Macau, Macau SAR 999078, P.R. China*

[b] *Zhuhai UM Science & Technology Research Institute, Zhuhai, Guangdong 519000, P.R. China*

[c] *Zhuhai Institute of Urban Planning & Design, Zhuhai, Guangdong 519000, P.R. China*

[d] *Guangdong Huazheng Construction Co., Ltd., Zhuhai, Guangdong 519000, P.R. China*

[e] *Zhuhai Transportation Holdings Group Co., Ltd., Zhuhai, Guangdong 519000, P.R. China*

[f] *CCCC Third Harbour Consultants Macau Co., Ltd., Macau SAR 999078, P.R. China*

[g] *Fujian Provincial Key Laboratory of Terahertz Functional Devices and Intelligent Sensing, School of Mechanical Engineering and Automation, Fuzhou University, Fuzhou, Fujian 350108, P.R. China*

* Corresponding authors. ** Corresponding authors. E-mail addresses: yc37801@um.edu.mo (Y. Chen), 974828540@qq.com (C. Tang), 569952523@qq.com (M, Cui), sunlong@cccltd.cn (L. Sun), mingliu@fzu.edu.cn (M.

Liu), gxsun@um.edu.mo (G. Sun). Present address: N23 University of Macau, Avenida da Universidade, Taipa, Macau, China

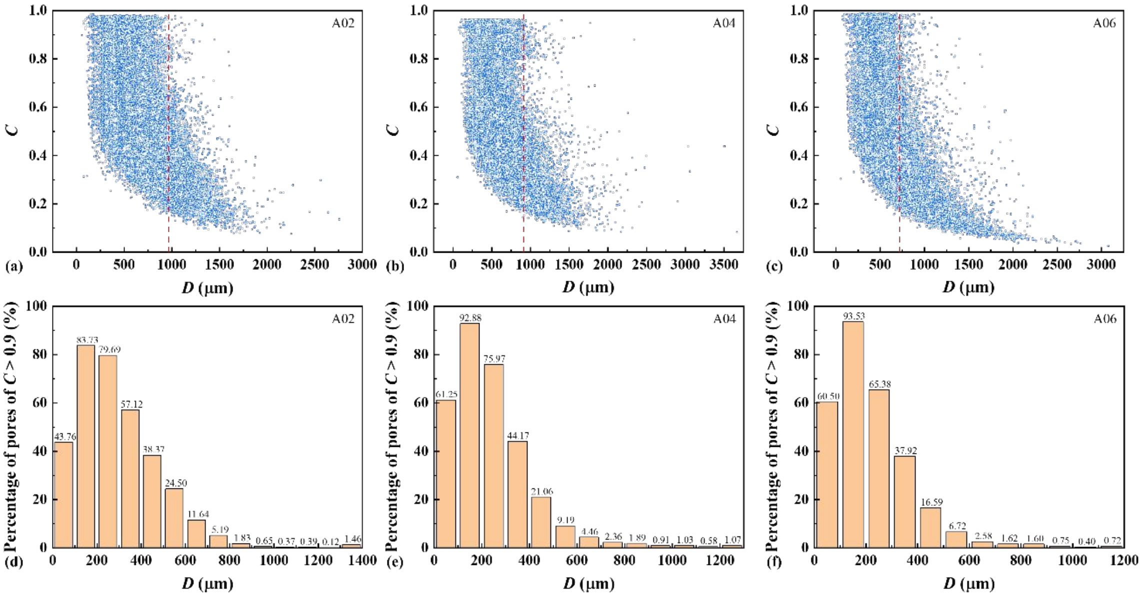


Figure S1. The correlation between pore diameter $D$ and circularity $C$ for A02, A04 and A06: $C$ vs. $D$ map (the red dash line divides the pores into two parts) see (a), (b) and (c); and the percentage of pores of $C > 0.9$ within different ranges of pore diameter $D$ see (d), (e) and (f).

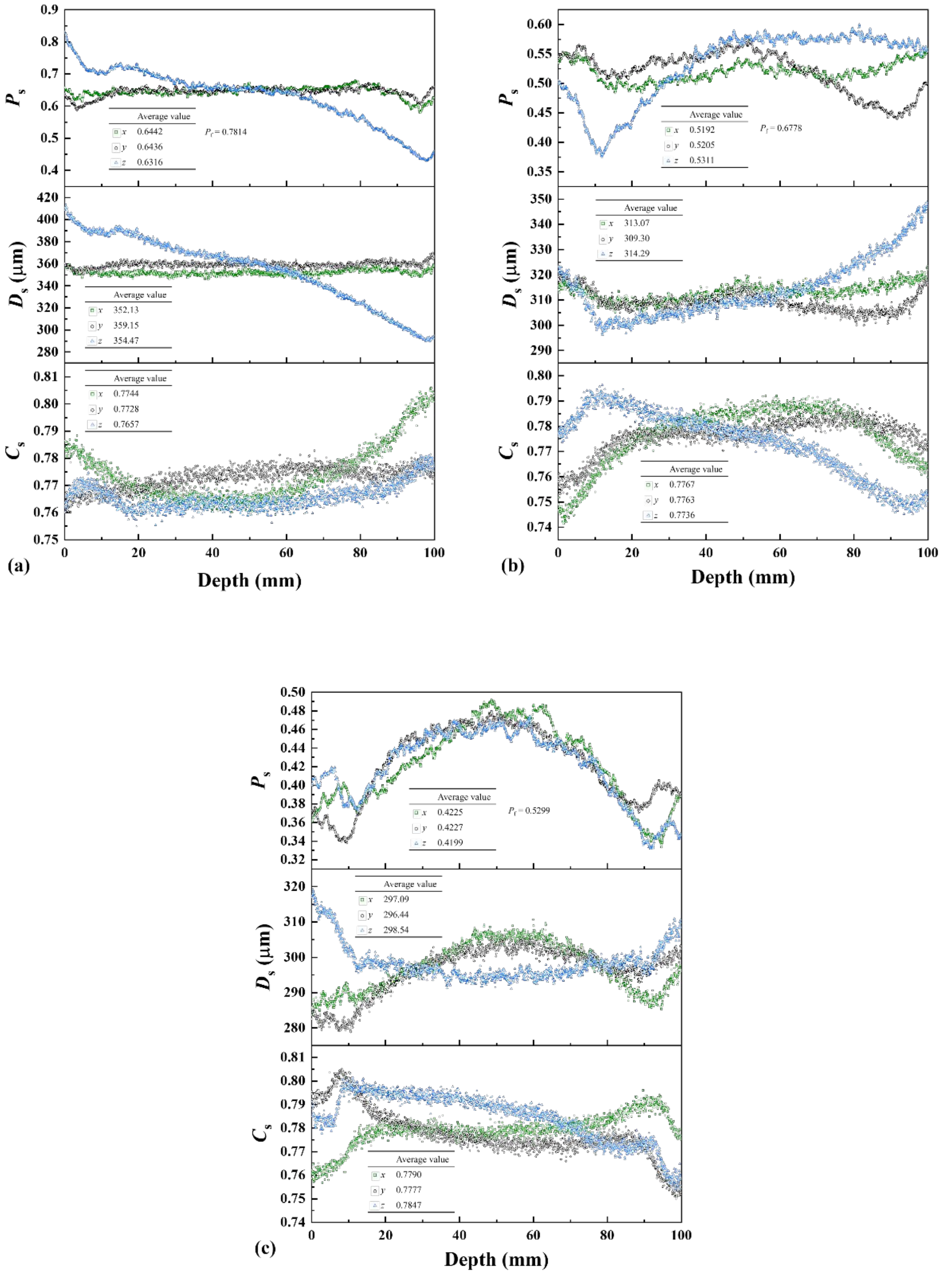


Figure S2. Variations of cross-sectional porosity $P_s$, average pore diameter $D_s$, and average circularity $C_s$ in $x$, $y$ and $z$ directions of foamed concrete of (a) A02, (b) A04 and (c) A06. The average values of $P_s$, $D_s$ and $C_s$ in per directions and the foam porosity $P_f$ calculated by Eq. (1) are listed as the insets.

$$P_f = 1 - \rho/\rho_0 \quad (1)$$
